# The Role of scaRNAs in Adjusting Alternative mRNA Splicing in Heart Development

**DOI:** 10.3390/jcdd5020026

**Published:** 2018-05-08

**Authors:** Chloe Nagasawa, Allison Ogren, Nataliya Kibiryeva, Jennifer Marshall, James E. O’Brien, Naoya Kenmochi, Douglas C. Bittel

**Affiliations:** 1 College of Biosciences, Kansas City University, Kansas City, MO 64106, USA; cnagasawa94@kcumb.edu (C.N.); allison.ogren@kcumb.edu (A.O.); 2 Ward Family Heart Center, Children’s Mercy Hospital, University of Missouri-Kansas City School of Medicine, Kansas City, MO 64108, USA; nkibiryeva@cmh.edu (N.K.); jamarshall@cmh.edu (J.M.); jobrien@cmh.edu (J.E.O.J.); 3 Frontier Science Research Center, University of Miyazaki, Miyazaki 889-1692, Japan; kenmochi@med.miyazaki-u.ac.jp

**Keywords:** alternative mRNA splicing, congenital heart defects, scaRNAs, small cajal body-associated RNAs, tetralogy of Fallot

## Abstract

Congenital heart disease (CHD) is a leading cause of death in children <1 year of age. Despite intense effort in the last 10 years, most CHDs (~70%) still have an unknown etiology. Conotruncal based defects, such as Tetralogy of Fallot (TOF), a common complex of devastating heart defects, typically requires surgical intervention in the first year of life. We reported that the noncoding transcriptome in myocardial tissue from children with TOF is characterized by significant variation in levels of expression of noncoding RNAs, and more specifically, a significant reduction in 12 small cajal body-associated RNAs (scaRNAs) in the right ventricle. scaRNAs are essential for the biochemical modification and maturation of small nuclear RNAs (spliceosomal RNAs), which in turn are critical components of the spliceosome. This is particularly important because we also documented that splicing of mRNAs that are critical for heart development was dysregulated in the heart tissue of infants with TOF. Furthermore, we went on to show, using the zebrafish model, that altering the expression of these same scaRNAs led to faulty mRNA processing and heart defects in the developing embryo. This review will examine how scaRNAs may influence spliceosome fidelity in exon retention during heart development and thus contribute to regulation of heart development.

## 1. Introduction

Congenital heart defects (CHD) constitute a major proportion of clinically significant birth defects worldwide [1,2]. For most cases of CHD, the underlying cause is unknown. In the last decade, efforts in trying to identify a genetic basis for CHDs have dramatically improved our understanding of vertebrate heart development but failed to elucidate the underlying genetic cause of most CHDs. During embryonic development spatiotemporal signaling between the first and second heart field, gives rise to the left and the right ventricle and conotruncal outflow tract which is critical for correct vertebrate heart development [3,4,5,6,7,8,9]. Studies have shown that mRNA splicing of the vertebrate heart is highly dynamic and intricately regulated during embryonic heart development [3,10,11,12], but no direct link has been shown between alternative splicing and CHDs. Vertebrate heart development is controlled by master regulatory genes (e.g., NKX2.5, GATA4, MBNL1, RBFOX2) that activate key developmental pathways, including WNT and NOTCH pathways. Almost all of the genes that are part of these pathways have known, alternatively spliced mRNA isoforms. Taken together, these observations suggest that if mRNA splicing is dysregulated it could contribute to development of a cardiac defect.

Mature messenger RNA is produced from eukaryotic genomes by removing the introns from almost all protein coding genes [13]. In addition, most eukaryotic genes have been shown to be alternatively spliced (~90%) [14,15], a process that plays a role in normal development as well as pathogenesis [3,16,17,18,19,20]. Pre-mRNA processing, or splicing, is carried out mostly by the spliceosome, a multimegadalton ribonuceloprotein complex made up of multiple proteins and five snRNAs (small nuclear RNAs: U1, U2, U4, U5, and U6). Research has shown that spliceosomal function and composition has been conserved in eukaryotes, therefore maintaining its importance throughout life [21]. Spliceosomal function is a multistep process: the reactive subgroups are aligned through RNA-RNA-protein interactions and rearranged as each intron is identified, the intron-exon boundaries are located, and finally each intron is removed from pre-mRNA through catalysis.

Alternative splicing creates different combinations of exons to increase the total number of all mRNAs and thereby increasing the number of proteins that can be manufactured by the cell. Although some exons are present in every mature mRNA, alternative splicing generates distinct variations of proteins that are expressed in different tissues and at different intervals in the same tissue. Recent studies have shown that alternatively spliced pre-mRNA follows a regulated trajectory during the transition from fetal to post-natal heart development in the mouse [22]. That evidence suggests that alternative splicing of mRNA plays a role in mammalian cardiac development, but the contribution to congenital heart defects remains unknown.

Although 80% of the human genome is transcribed, only 2% is actually translated into protein [23]. Recent evidence has shown that noncoding RNAs (ncRNA) are important for proper heart development through proper spatiotemporal expression of specific microRNAs [24]. A class of ncRNAs, the small nucleolar RNAs (snoRNAs), have been evolutionarily conserved in all eukaryotes. Their canonical function is to facilitate biochemical modification of specific nucleotides (i.e., methylation and pseudouridylation) of ribosomal RNAs and spliceosomal RNAs (snRNAs, also referred to as small nuclear RNAs). A class of snoRNAs called scaRNAs (small cajal body specific RNAs) target the snRNAs that are centrally important for the function of the spliceosome [25].

The cornerstone of our initial studies was the analysis of the transcriptome of right ventricular tissue from infants with tetralogy of Fallot (TOF). Our studies showed scaRNAs and their target snRNA were reduced in heart tissues from infants with TOF relative to heart tissue from normally developing infants [26]. Those studies also revealed that mRNA splicing in the right ventricular tissue of infants with TOF were significantly different when compared with the control of tissue from normally developing infants. We therefore hypothesized that reduced levels in scaRNAs could have an impact on spliceosome function, resulting in dysregulation of mRNA splicing that could in turn impair heart development. It seems plausible that alternative isoforms of components that make up the regulatory networks that govern heart development could alter the spatiotemporal signaling between the first and second heart fields, resulting in abnormal conotruncal alignment.

Without biochemical modification, spliceosome function will be compromised [27,28]. The role that scaRNAs play in development as a consequence of their role in spliceosomal maturation is currently unclear. However, there is ample evidence to suggest that scaRNA expression, as well as mRNA alternative splicing is tissue specific and developmentally regulated. We used human primary cells from the right ventricle of infants with TOF and from normally developing infant heart tissue to investigate the impact of scaRNA expression level on mRNA splicing in vitro. We also altered scaRNA levels in zebrafish embryos to assess the impact of altered scaRNA expression on mRNA splicing and development in vivo.

## 2. Material and Methods

### 2.1. Subjects

Our subjects were children less than one year of age with CHDs: TOF, Transposition of the Great Arteries (TGA), or pulmonary atresia with intact ventricular septum (PA/IVS) requiring surgical reconstruction. Informed consent was obtained from a parent or legal guardian after reviewing the consent document and having their questions answered (IRB # 11120627). Our acquisition and characterization of control human infant heart tissues and human fetal heart tissue has been previously described [26,29]. Our original observations were based on analysis of tissue from 16 infants with idiopathic TOF (nonsyndromic, without 22q11.2 deletions, 11 males, 5 females), comparison tissues from eight normally developing infants (3 males, 5 females). The diagnosis and anatomy were obtained by echocardiography and angiography, and confirmed at the time of surgery. All microarray analyses were run on samples from 16 infants with nonsyndromic TOF (i.e., no 22q11.2 deletion), 8 infants with normally developing hearts, and 3 fetal samples (Table 1). We also recruited an additional 8 subjects with nonsyndromic TOF to allow for independent validation via quantitative reverse transcription polymerase chain reaction (RT-PCR).

We obtained 3 fetal hearts (~90 days gestation) through the National Institute of Child Health and Human Development supported tissue retrieval program from the Central Laboratory for Human Embryology at the University of Washington (Seattle, WA, USA). The fetal hearts were dissected by one of the surgeons who also performed many of the reconstructions of the conotruncal defects (J.E.O.J.), to ensure the tissue analyzed was from a similar location as the tissues removed during surgery. Comparison tissues from 8 normally developing infants (3 males, 5 females) were obtained from LifeNet Health (http://www.lifenethealth.org). LifeNet Health is a nonprofit regenerative medicine company that provides bio-implants and organs for transplantation. The control subjects were matched for age to the study population, and all control subjects expired owing to noncardiac-related causes. LifeNet Health follows the following protocol for tissue recovery: (1) If the donor is placed in a refrigerated morgue within 12 h of asystole, tissues can be recovered for up to 24 h and placed in a 1 °C to 10 °C sterile isotonic solution; or (2) if the donor is not refrigerated, tissues can be recovered for up to 15 h and placed in a 1 °C to 10 °C sterile isotonic solution. All donor tissue was de-identified, no donor confidential information was disclosed, and consent was obtained to use the tissue for research. In addition, we have now characterized scaRNA, spliceosomal RNAs and the splicing pattern of six key transcription factors from an additional 21 infants with idiopathic TOF, all of whom met the same criteria as our original subjects.

### 2.2. Derivation of Primary Cells

Tissue samples were collected at the time of surgical correction of the defect. Detailed subject descriptions were previously published [26,29]. Primary cell cultures were derived from RV tissue of infants with TOF. The RV tissue was immediately immersed in DMEM (Invitrogen/Gibco, Grand Island, NY, USA) plus 10% fetal calf serum (Sigma/Safc, St. Louis, MO, USA) and 1% penn/strep (Invitrogen/Gibco, Grand Island, NY, USA). The tissue was minced and most of the media was removed, leaving only enough to keep the tissue from drying out. After 24 h, additional media was added and cells were growing robustly after 3 to 4 days. Media was exchanged every 48 h. In addition, we obtained a primary neonatal cardiomyocyte cell culture derived from normally developing human neonatal cardiac tissue from Celprogen (Cat#36044-21, San Pedro, CA, USA). These cells were also grown in DMEM plus 10% fetal calf serum and 1% penn/strep.

### 2.3. Transfection of scaRNA Plasmids into Primary Cells

The expression vectors pCGL-SCARNA4 (ACA26) and pCGLSCARNA1 (ACA35) were a generous gift from Dr. Tamas Kiss, Universite Paul Sabatier [30]. We used ACA26 and ACA35 here to maintain continuity with previous work. The scaRNAs were cloned into an intron sequence between hemoglobin exons 3 and 4 so that they would be correctly processed in vivo and expression was driven with the CMV promoter. We replaced ACA35 with the corresponding sequences from the scaRNAs: SNORD94, SCARNA8, SNORD67, and SCARNA23. The scaRNAs were transfected into the primary cell lines derived from infants with TOF according to the manufacturer’s protocol. Briefly, 2 μg of plasmid DNA was diluted in 200 μL of serum free media and added to 2 μL of the Poly Magnetofectant (a magnetic nanoparticle transfection reagent; OZ Biosciences INC. USA, San Diego, CA USA), vortexed and incubated for 20 min at room temperature. The transfection mixture was added dropwise to 2 × 105 cells in 1.8 mL of media containing 10% serum in a single well of a 6-well plate. The culture plate was set on top of a plate magnet (OZ Biosciences) for 20 min, and returned to the incubator. After 72 h, the cells were trypsinized, pelleted and stored at −80 °C until processed for RNA extraction.

### 2.4. scaRNA Knockdown

We used antisense LNA oligos (locked nucleic acid oligos, Exiqon Life Sciences, Woburn, MA, USA) to suppress the scaRNAs in primary cardiomyocytes as has been done previously in immortalized cell cultures [22]. Briefly, the LNA oligo protocol is as follows, 50 μM LNA oligo in 100 μL serum free media is mixed with 12 μL HiPerfect transfection reagent (Qiagen, Valencia, CA, USA) and incubated for 20 min at room temperature. The transfection mixture was added to 2 × 105 cells in 2.3 mL of media with 10% serum in a single well of a 6-well cell culture plate. After 48 h, the cells were pelleted and stored at −80 °C until processing. Identifying LNA oligos for effective knockdown of the scaRNA was an empirical process. Two to four oligos were tested for each scaRNA to determine which were most effective at knocking down the target scaRNA.

### 2.5. RNA Isolation and qRT-PCR (Human Tissue)

RNA was extracted from ~2 × 106 cells using a mirVana miRNA isolation kit (Invitrogen, Grand Island, NY, USA) according to the manufacturer’s instruction. Briefly, an equal quantity of total RNA (1 μg) together with random and oligo dT primers was reverse transcribed using Superscript III (Invitrogen by Life Technologies, Carlsbad, CA, USA) according to the manufacturer’s directions. Quantitative RT-PCR (qRT-PCR) was performed using Power SYBR Green PCR Master Mix (Applied Biosystems, Foster City, CA, USA) according to the manufacturer’s directions as previously described [29]. The reaction was carried out in an ABI7000 system (Applied Biosystems, Foster City, CA, USA) beginning with 10 min at 95 °C. The intensity of the SYBR Green fluorescence was measured at the extension step of each cycle. At least three replicates were performed on each sample for each gene.

### 2.6. Microarray Analysis of Splice Variants

The exon arrays were AffymetrixHuEx-1_0-st-v2. The raw data for the arrays have been deposited in the Gene Expression Omnibus (miRNA arrays accession No. GSE35490) as described previously [26]. All arrays were run at the University of Kansas Medical Center-Microarray Facility (KUMC-MF) according to the manufacturer’s protocols. KUMC-MF is supported by the University of Kansas, School of Medicine, KUMC Biotechnology Support Facility, the Smith Intellectual and Developmental Disabilities Research Center (HD02528), and the Kansas IDeA Network of Biomedical Research Excellence (RR016475). All statistical analyses were performed using statistical software: Partek Genomics Suite software version 6.6 (Partek Inc., Chesterfield, MO, USA), and Bioinformatic assessment was done using Ingenuity Pathways Analysis (IPA, Ingenuity Systems, Inc., Redwood City, CA, USA). Raw data (CEL. files) were uploaded into Partek Genomics Suite for normalization and statistical analysis. Robust Multichip Analysis (RMA) was used for background correction, followed by quintile normalization with baseline transformation to the median of the control samples. Only probes with intensity values >20% of background value, in at least 1 of the conditions, were included for additional analysis. A Student *t*-test with a Benjamini and Hochberg multiple test correction for false discovery rate (FDR) was used to determine significance. Probes were filtered using an FDR adjusted *p*-value ≤ 0.05. IPA was used to assess networks, functions, and/or canonical pathways represented by the list of alternatively spliced genes. Fisher’s exact test was used to identify the most significantly (*p* ≤ 0.05) altered biological functions and/or disease categories within the dataset.

### 2.7. Zebrafish

Zebrafish (*Danio rerio*, wild-type AB line) were raised and maintained under standard laboratory conditions at the Division of Bioresources, Frontier Science Research Center, University of Miyazaki, Japan. We targeted orthologous scaRNAs in zebrafish for knockdown with antisense morpholinos designed to inhibit scaRNA processing as previously described [31]. A database search for the 12 scaRNAs that were reduced in TOF revealed that 7 have homologs in zebrafish, including scarna1, scarna8, scarna13, scarna14, and scarna2 that carry out U2 snRNA modifications, and snord94 and snord7 which carry out U6 snRNA modifications. These “shared scaRNA targets” ensure the comparability and relevance of the data obtained from the zebrafish experiments to human heart development. We chose to focus on two scaRNAs, scarna1 that targets U2 and snord94 that targets U6, as these are representative scaRNAs that had an impact on snRNA function when targeted in the human primary cell cultures.

### 2.8. Morpholino Oligonucleotide Injections and Morphological Analysis

Morpholino antisense oligos (MOs) to inhibit precursor scaRNA processing were obtained from Gene Tools, LLC (Philomath, OR, USA). The ACA35 (scaRNA1) and U94 (snord94) MOs were designed at the 3′ end of scaRNA within the fifth intron of ppp1r8b and second intron of *rrm2* gene, respectively. As a control, morpholinos with five mispaired bases (misMOs) were used. Based on our previous methods [31], the MOs were injected into the blastomere of one-cell stage embryos using an IM-30 Electric Microinjector (Narishige, Japan) at the following concentrations: U94MO at 5 μg/μL; and ACA35MO at 10 μg/μL. The control MOs were injected using the same volume. We examined the effects of scaRNA suppression on heart development in zebrafish embryos by microscopic observation. The morphology and physiological function of zebrafish heart in morphants were analyzed by live-video imaging.

### 2.9. RNA-Seq Analysis

Messenger RNA was extracted from zebrafish embryos at 6 and 24 hpf from wild type embryos and embryos injected with antisense or mismatch morpholinos. Sequencing was done using an Illumina^®^ Genome Analyzer™. Single-end mRNA-Seq reads were mapped to the zebrafish genome (danRar7), allowing up to two mismatches, using the STAR aligner and the Partek Flow™ interface (Partek Inc. St. Louis, MO, USA). The output BAM files were imported directly into Partek Genomics Suite 6.6 (PGS, Partek Inc. St. Louis, MO, USA) and analyzed using the RNA-Seq interface. To detect differentially spliced genes, mRNA quantification was performed using the reads per kilobase of exon per Million mapped reads (RPKM) model for normalization [24]. Reads were assigned to individual transcripts/exons of a gene based on the Expectation/Maximization (E/M) algorithm [31] using the ENSEMBL database. Nevertheless, annotation of the zebrafish genome is limited with respect to splicing variants, so extensive analysis of genome-wide splicing was inadequate. We therefore, focused on members of the WNT pathway which is more extensively annotated and also critical for regulating heart development. We evaluated exon retention in those genes using PGS ANOVA with a significant *p*-value ≤ 0.05.

## 3. Results and Discussion

Twelve scaRNAs are reduced in the right ventricle of infants with TOF and these 12 scaRNAs target only U2 and U6.

We identified 12 scaRNAs with reduced expression in heart tissue from infants with TOF (Table 1 [26]), the nucleotides targeted for modification by the 12 scaRNAs reduced in TOF through the scaRNABase database (http://www-scarna.biotul.fr/index.php). We found that the only two snRNAs predicted to be targeted were U2 and U6. Within U2, six of the scaRNAs targeted 10 nucleotides (of the 23 nucleotides known to be modified by scaRNAs). Within U6, six scaRNAs targeted 5 nucleotides (of 8 total known modified nucleotides). Our original observations were based on analysis of heart tissue from 16 subjects. In addition to suppression of scaRNA expression, both U2 and U6 had significantly reduced expression in the original 16 TOF samples as compared to the 8 controls (U2 was reduced 1.8-fold in TOF RV, *p* = 0.04, and U6 was reduced 3.2-fold in TOF RV, *p* < 0.0001). These results suggest U2 and U6 may have reduced stability without efficient scaRNA biochemical modification.

### 3.1. Cardiac Regulatory Networks Are Enriched for Alternative Splice Isoforms in TOF

We have analyzed tissue from an additional 24 infants with TOF (for a total of 40 subjects analyzed) which corroborates our original observations. All of these additional subjects analyzed had scaRNA levels and alternative splicing that were consistent with our previous analyses. While these analyses are not shown here, these additional analyses underscore the consistency of the scaRNA expression and alternative splicing of index genes in RV tissue from infants with TOF.

**Figure 1:**
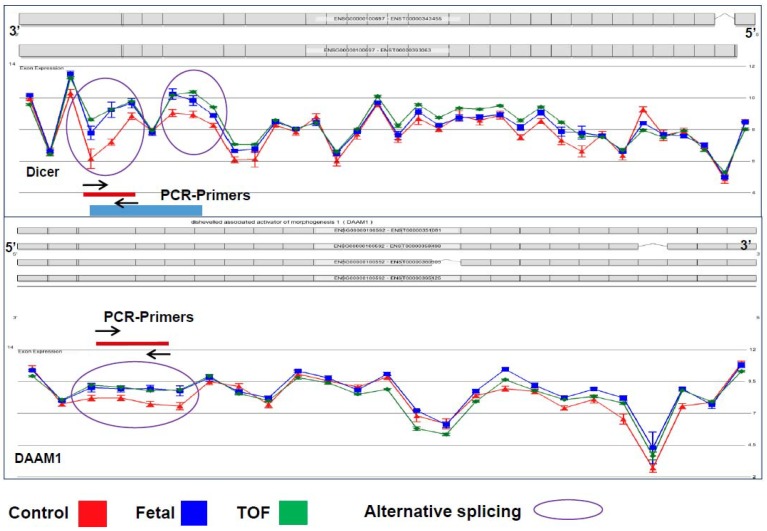
Representative examples of alternative splicing of two genes (DICER1 and DAAM1). Circled regions represent exonic regions where multiple probes had a significant change in probe intensity level between the normally developing and control subjects and the subjects with TOF. Note that there is not a significant difference in the probe intensities in other regions of the genes. In addition, note that the blue line represents probe intensities of samples from fetal tissue which differed significantly from the control tissue in the circled regions but did not differ from the samples from subjects with TOF. Standard error bars are shown for each probe. *N*: TOF = 16, Control = 8, Fetal = 3.

Coordinated control of alternative splicing plays a role in most developmental processes through modifying the transcriptome and thus the proteome. Appropriate splicing has been shown to be important for proper heart development in multiple animal model studies, but the underlying sequence of events involved in tissue-specific alternative splicing is still poorly understood [10,12,22]. Chiefly, the significance of scaRNA guided nucleotide modification in spliceosomal RNAs has not been investigated, nor the potential ramifications on transcript splicing. Our analyses of splicing variants in TOF myocardium showed an increase of alternative transcript isoforms within gene networks known to be involved in essential regulation of heart development, such as the WNT and NOTCH pathways. Remarkably, normal fetal RV tissue contained >50% of these isoforms, suggesting that mRNA splicing failed to follow its normal progression during heart development in infants with TOF. We will refer to these alternatively spliced forms as fetal-type mRNA isoforms from this point on. Figure 1 depicts two representative examples of alternative splicing, DICER and DAAM1, which have similar splicing patterns in fetal and TOF tissues (blue and green lines) compared to the control (red line). Dicer has been shown to be essential in mouse heart development, particularly playing a role in splicing regulation [10]. Recently, variants of Daam1, a member in the WNT pathway, have also been shown to have an impact on angiogenesis and endothelial cell migration and tube formation [32].

### 3.2. Primary Cell Lines Derived from TOF Myocardium Retain the Same Relative Expression Patterns as the Tissue

We have derived primary cell lines from right ventricular myocardium obtained from 15 infants with TOF (TOF primary cells—TOFpc). We compared scaRNA level, U2 and U6 level, and splice isoform patterns between TOFpcs and primary myocytes cells derived from normally developing infant heart tissue. The fetal type pattern of scaRNA spliceosomal RNA expression and spliceosome isoforms of index genes was retained in TOFpcs, relative to cells derived from normally developing neonatal heart tissue (data not shown). TOFpcs also retained a splicing pattern consistent with the TOF pattern relative to control, and these changes were maintained through at least four passages of the cell lines.

### 3.3. Overexpression of ACA26 and ACA35 in TOF Primary Cells Was Associated with an Increase in U2 Levels and a Decrease in Fetal Splice Isoforms

In order to ensure correct processing in vivo, the scaRNAs were cloned into an intron between hemoglobin exons 3 and 4, and the CMV promoter was used to drive expression [33]. Increases in scaRNA were observed when either of the plasmids were transfected alone but there was no change in U2 level or in splicing (data not shown). However, simultaneous transfection of the plasmids pCGL-ACA26 and pCGL-ACA35 into TOF primary myocytes produced a modest (~50%) but significant upregulation in U2 level, suggesting scaRNA-directed modification of snRNAs is necessary for snRNA stability.

**Figure 2:**
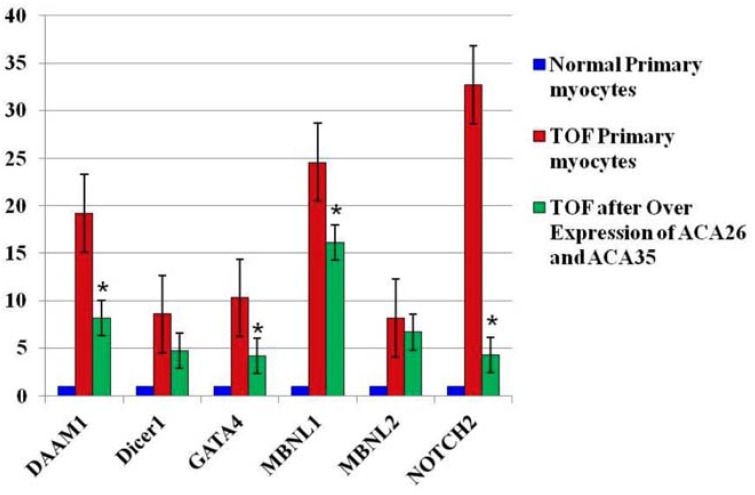
Fetal-type splice isoforms are reduced after overexpression of scaRNAs. Data are fold change of exons that represents regions of variability within the alternatively spliced gene. Each value is derived from the mean intensity level from three different primary myocyte cell lines (3 biological replicates with 3 technical replicates for each individual gene analyzed by qRT-PCR). The normal comparison cells (blue) provide a reference point for comparisons between the sham transfected TOF cells and those with the expression vectors. * significantly different from levels in sham transfected TOF primary cells. The TOF primary cells were derived from three different infants and each PCR included 3 technical replicates. Expression plasmids pCGL-ACA26 and pCGL-ACA35 were a generous gift from Dr. Tamas Kiss, Universite Paul Sabatier, Toulouse, France.

Importantly, the fetal-type splice forms were reduced in the 6 index genes that we monitored (Figure 2). These data were repeated in TOFpcs from three different infants (genotypes). Thus, these observations support our hypothesis that the scaRNAs participate significantly in regulating spliceosome function, thus contributing to regulation of mammalian heart development.

### 3.4. Knockdown of scaRNAs (ACA35 Targeting U2, or U94 Targeting U6)

As shown in studies of snoRNA-directed modification of ncRNA in archaea and lower eukaryotes, nucleotide modifications are essential for ncRNA function [34,35]. Similar conclusions in vertebrates had not been described until Kenmochi and colleagues established the developmental significance of snoRNAs in zebrafish [31]. They suppressed the expression of several snoRNAs and using a novel mass spec analysis, they demonstrated that decreased snoRNA expression reduced the snoRNA-guided methylation of target nucleotides. The loss of rRNA nucleotide modification, even at a single site, caused morphological defects and embryonic lethality in zebrafish. Their observations suggest that snoRNA guided modification of rRNA plays an important role in regulating vertebrate development.

**Figure 3:**
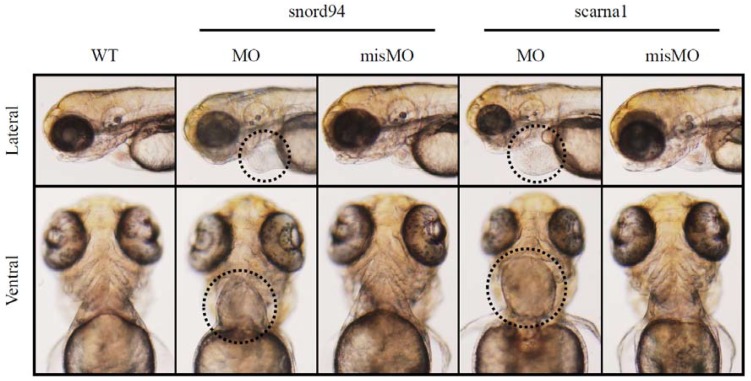
Representative images of zebrafish wildtype embryos (WT) and embryos treated with antisense morpholinos (MO) and mismatch morpholinos (misMO). Ventral view. The heart is circled in the morphants treated with antisense morpholinos.

We suppressed the expression of two scaRNAs, ACA35 and U94 (both are homologous to scaRNAs identified by our screens of TOF myocardium) in zebrafish. Each scaRNA knockdown produced developmental abnormalities, including heart malformations (Figure 3). This is a compelling finding when taken together with our observations that scaRNA levels affect splicing in TOF primary cardiomyocytes, suggesting that scaRNAs have an essential, although still unrecognized, role in vertebrate heart development. Interestingly, these specific scaRNAs appear to primarily affect heart development.

### 3.5. Splice Isoforms Change during Development in Zebrafish and after Targeted Knockdown of scaRNAs

We downloaded RNA-Seq data from the Gene Expression Omnibus derived from developing zebrafish at 0.75 h, 6 h, 1 d, 2 d, 3 d, and 5 d post fertilization (GEO#: GSE30603) and analyzed for alternative splicing. A large proportion of genes showed clear changes in ratios of splice isoforms, including genes essential for cardiac development (e.g., Gata4, Mbnl1, Notch1, Dicer, data not shown).

**Figure 4:**
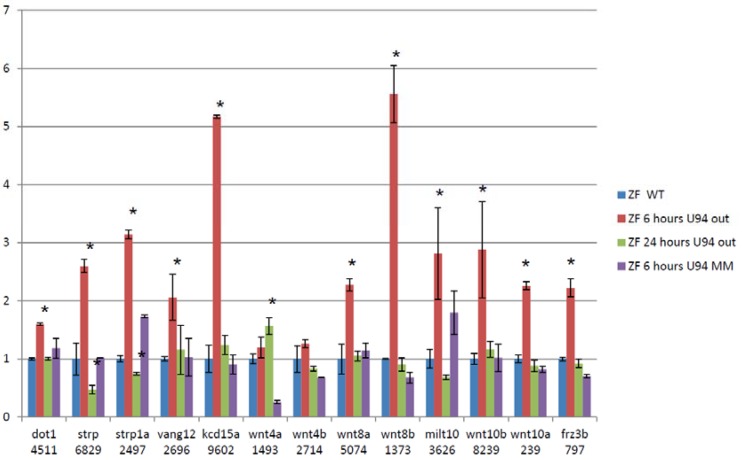
Treating zebrafish embryos with Anti-U94 morpholino causes changes in exon retention of cardiac regulatory genes. 13 of 39 members of the WNT family have changes in exon retention after treatment with antisense morpholino (assessed by RNA-Seq and qRT-PCR, Values shown are from qRT-PCR data). Data are fold change of exons that represents regions of variability within the alternatively spliced gene. (Each gene analyzed by qRT-PCR was done on 3 separate embryo samples with 3 technical replicates). * significant p < 0.05.

We then performed RNA-Seq on RNA extracted from 24 hpf zebrafish embryos treated with antisense morpholinos directed at ACA35 or U94 and WT untreated embryos and embryos treated with mismatch morpholinos. Paired-end sequencing runs were performed with 101 base reads on the Illumina HiSeq 1500. RNA-Seq data were analyzed using the “Tuxedo Suite.” No significant differences were shown between mismatch morpholino and WT embryos. U94 was reduced by approximately 40% and ACA35 was not detectable in their respective knockdown morphants compared to WT or mismatch treated embryos. An example of alternative splicing for several members of the WNT pathway is shown following U94 knockdown (Figure 4). These analyses demonstrate the dynamic nature of splicing in developing zebrafish, similar to that of mammals. Collectively, these experiments firmly support our hypothesis that scaRNAs play an important role in regulating mRNA splicing variants which are critical for heart development.

## 4. Conclusions

We investigated the transcriptome in myocardial tissue from children with tetralogy of Fallot (TOF) and observed changes in mRNA splice isoforms of genes that are essential for regulating heart development [26]. Similarly, we found a significant reduction in the levels of 12 small cajal body specific RNAs that are important for maturation of spliceosomal RNAs. We manipulated the expression of two scaRNAs in vitro (cell cultures) and in vivo (zebrafish). We observed marked changes in splicing patterns, and especially, developmental deficiencies including heart defects in zebrafish when scaRNA expression was reduced. Although our experiments with zebrafish cannot recapitulate tetralogy of Fallot since the fish have only have a two chambered heart, our findings support a crucial role for scaRNAs in maintaining the fidelity of the spliceosome. We hypothesize that disruption of normal mRNA splicing due to loss of spliceosome fidelity could disturb transitions of mRNA isoforms that occur during vertebrate heart development that would likely alter regulatory processes leading to defective heart development. These observations illuminate a new paradigm in developmental regulation and could help explain some of the missing genetic heritability of CHDs. Moreover, we suggest that our data are supportive of a ubiquitous mechanism that represents a new paradigm in developmental regulation.

## Figures and Tables

**Table 1:** 12 scaRNAs with reduced expression in heart tissue from infants with TOF.

Affy Probe Set ID	Target	Chromosome	Alternate Name	Host Gene
HBII-382_s_st	U2 snRNA C61 and U2 snRNA G11	1		
mgU2-19-30	U2 snRNA G19 and U2 snRNA A30	X	scaRNA9	
mgU2-25-61_st	U2 snRNA G25 and U2 snRNA C61	1	scaRNA2	
U92_st	U2 snRNA U34 and U2 snRNA U44	9	scaRNA8	FAM29A
ACA26_st	U2 snRNA U41 and U2 snRNA U39	1	scaRNA4	KIAA0907
ACA35_st	U2 snRNA U89	1	scaRNA1	PPP1R8
mgU6-47_st	U6 snRNA A47	17	SNORD7	
mgU6-53_st	U6 snRNA A53	14	SNORD8	CHD8
mgU6-53B_st	U6 snRNA A53	14	SNORD9	CHD8
HBII-166_st	U6 snRNA C60	11	SNORD67	CKAP5
U94_st	U6 snRNA C62	2	SNORD94	PTCD3
ACA12_st	U6 snRNA C62	X	scaRNA24	POLA1
